# FDG-PET/CT imaging for evaluating durable responses to immune check point inhibitors in patients with advanced cutaneous squamous cell carcinoma

**DOI:** 10.1186/s40644-021-00426-2

**Published:** 2021-10-13

**Authors:** Luke S. McLean, Karda Cavanagh, Rodney J. Hicks, Jason Callahan, Jing Xie, Anthony Cardin, Annette M. Lim, Danny Rischin

**Affiliations:** 1 Department of Medical Oncology, Peter MacCallum Cancer Centre, Melbourne, Victoria Australia; 2 Cancer Imaging, Peter MacCallum Cancer Centre, Melbourne, Australia; 3 University of Melbourne, Parkville, Melbourne, Australia; 4 Biostatistics and Clinical Trials, Peter MacCallum Cancer Centre, Melbourne, Australia

**Keywords:** Cutaneous cancer, Immunotherapy, FDG-PET, PERCIST1.0, RECIST1.1

## Abstract

**Background:**

The role of FDG-PET/CT imaging in assessing response to immunotherapy in advanced cutaneous squamous cell carcinoma (CSCC) is unknown. This study compared complete metabolic response (CMR) rates by FDG-PET and RECIST1.1 via CT or MRI in patients on cemiplimab for > 10 months.

**Methods:**

This was a single-centre retrospective study of 15 patients treated with cemiplimab for advanced CSCC who had CT/MRI and FDG-PET/CT at > 10 months to assess metabolic treatment response. The median age was 73 years (range 55–84) and 93% were male. RECIST1.1 and PERCIST1.0 tumor responses were evaluated by blinded readers.

**Results:**

Seventy-three percent (11/15) (95%CI 44.9, 92.2%) achieved a CMR on PET. Of these 11, on RECIST1.1 there was one complete response, 9 partial responses and one stable disease.

**Conclusions:**

In patients on cemiplimab for > 10 months, there was discordance between CR rates on FDG-PET versus RECIST1.1. FDG-PET/CT may have utility for clarifying depth of response in patients treated with immunotherapy for CSCC.

## Background

Immunotherapy, such as cemiplimab and more recently pembrolizumab, has revolutionised the management of metastatic or locally advanced CSCC not amenable to curative surgery or radiation [1, 2]. The two single-arm immunotherapy trials which have led to regulatory approval utilised RECIST1.1 and WHO response assessments based on computed tomography (CT) or magnetic resonance imaging (MRI) [3]. However, no data exists regarding the role of 2-deoxy-2-[^18^F]fluoro-D-glucose (*FDG*)-positron emission tomography (PET) in CSCC immunotherapy treated cohorts despite FDG-PET/CT demonstrating improved ability to detect complete metabolic response (CMR) in melanoma patients treated with immunotherapy [4]. We observed that the majority of our long-term responding patients remained partial responders by RECIST1.1, with residual masses. We hypothesised that many of these long-term partial responders may have achieved a CMR on FDG-PET/CT imaging.

## Methods

This study was a single-centre retrospective review of FDG-PET/CT assessment of response to immune checkpoint inhibitors (ICI) in patients with advanced CSCC defined as locally advanced not amenable to curative surgery and/or radiotherapy or metastatic disease. Patients were treated on the NCT02760498 groups 1–3 and 5 [1, 5, 6] and were enrolled between January 2016 and July 2020, to receive cemiplimab with regular CT+/−MRI response assessments (every 8 weeks or 9 weeks (group 3)) but with no formal requirement for FDG-PET/CT evaluation. However, for inclusion in our study, we identified patients who had FDG-PET/CT imaging performed at least 10 months after starting treatment with correlating CT/MRI within 6 weeks. Blinded retrospective reviews of FDG-PET/CT as per PERCIST1.0 [7] and CT/MRI as per RECIST1.1 occurred by a nuclear medicine specialist and radiologist, respectively. On an audit of advanced CSCC patients presented in our multidisciplinary meeting over a 10 month period we found 71/72 patients had FDG-avid disease at baseline. Of the patients on cemiplimab included in this study two out of 15 patients didn’t have a baseline FDG-PET, however, given this finding were considered to have FDG-avid disease at baseline for the purposes of PET response assessment. Data collected included: age, gender, ECOG performance status, survival status, RECIST1.1 response and FDG-PET metabolic response as per PERCIST1.0. This study was approved by the Human Research Ethics Committee of the Peter MacCallum Cancer Centre, Melbourne, Australia with a waiver of consent granted. Twenty-four months of treatment was planned in all cohorts, except Group 3 where treatment could be ceased at 12 months.

Descriptive statistics for baseline characteristics of patients were reported. Continuous variables were described as mean, standard deviation, median, interquartile range, minimum and maximum, and qualitative variables were described as counts and percentages. CMR rate and 95% confidence interval (CI) were reported using probabilities of the binomial distribution. All statistical analyses were performed in R version 3.6.3 using standard and validated statistical procedures.

## Results

Fifteen patients were identified with imaging in the required timeframe. The median age was 73 years (range 55–84) and the majority of patients were male. Basic demographics are summarised in Table 1.

**Table 1:** Baseline patient and tumor characteristics

Characteristic	Total (***n*** = 15)
**Age, (years)**	
Mean (SD)	71.5 (8.8)
Median [range]	73.0 [55.0–84.0]
IQR	65.0–78.0
**Sex, n (%)**	
Female	1 (7%)
Male	14 (93%)
**ECOG at treatment, n (%)**	
0	7 (47%)
1	8 (53%)
**Duration of cemiplimab treatment (months)**	
Median (range)	22 (8–24)

**Fig. 1:**
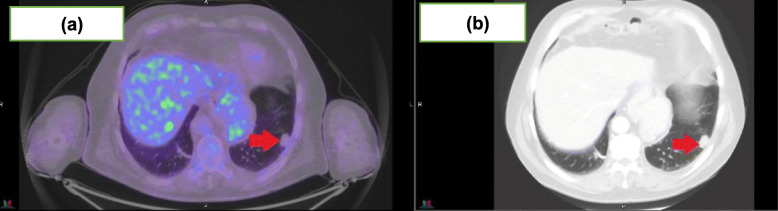
73 year old man with imaging for metastatic cutaneous squamous cell carcinoma to the lung post 12 months of cemiplimab 350 mg/3-weekly. **a** demonstrating a CMR to treatment and (**b**) demonstrating ongoing RECIST1.1 PR with measurable disease on CT

**Fig. 2:**
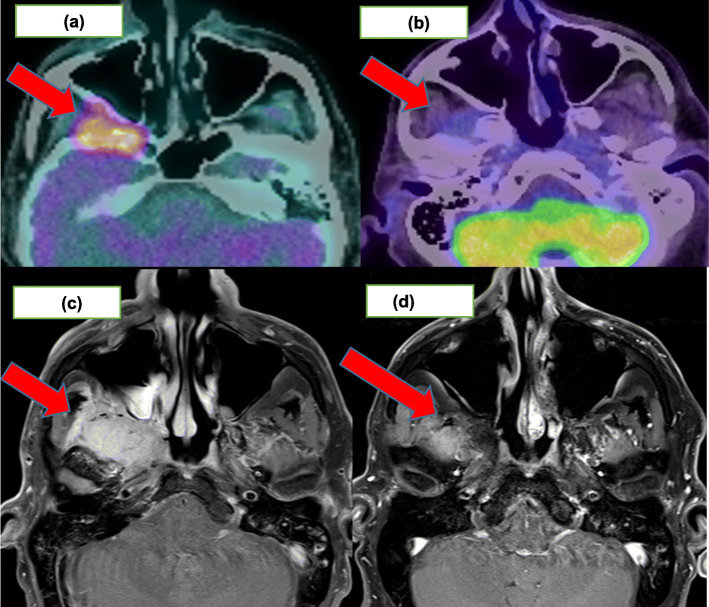
68 year old man with imaging for advanced cutaneous squamous cell carcinoma involving the right skull base, right infratemporal fossa and right trigeminal nerve managed with cemiplimab 350 mg/3-weekly. **a** demonstrating avid disease at baseline and (**b**) a CMR post 24 cycles. **c** demonstrating 41 × 23 mm enhancing mass on baseline MRI and (**d**) an ongoing RECIST1.1 PR post 24 cycles

Using FDG-PET, 11 patients achieved a CMR (73% [95%CI:44.9, 92.2%]) of which only one achieved a complete response (CR) on RECIST1.1 assessment. Nine (82%) of these 11 patients had a partial response (PR) on RECIST1.1 for which two examples are shown in Figs. 1 and 2. Response assessments for FDG-PET and RECIST1.1 were concordant for the two patients with progressive disease. Table 2 highlights the imaging responses for this cohort. Cohen’s Kappa value for overall concordance between RECIST1.1 and PERCIST1.0 evaluation was − 0.18.

**Table 2:** PERCIST1.0 versus RECIST1.1 response

Characteristic	PERCIST1.0 response			Total (***n*** = 15)
	CMR (***n*** = 11)	SMD (***n*** = 2)	PMD (***n*** = 2)	
**RECIST1.1 response, n(%)**				
CR	1 (9%)	0	0	1 (7%)
PR	9 (82%)	2 (100%)	0	11 (73%)
SD	1 (9%)	0	0	1 (7%)
PD	0	0	2 (100%)	2 (13%)

*CMR* complete metabolic response, *CR* complete response, *PMD* progressive metabolic disease, *PMR* partial metabolic response, *PR* partial response, *SD* stable disease, *SMD* stable metabolic disease

**Fig. 3:**
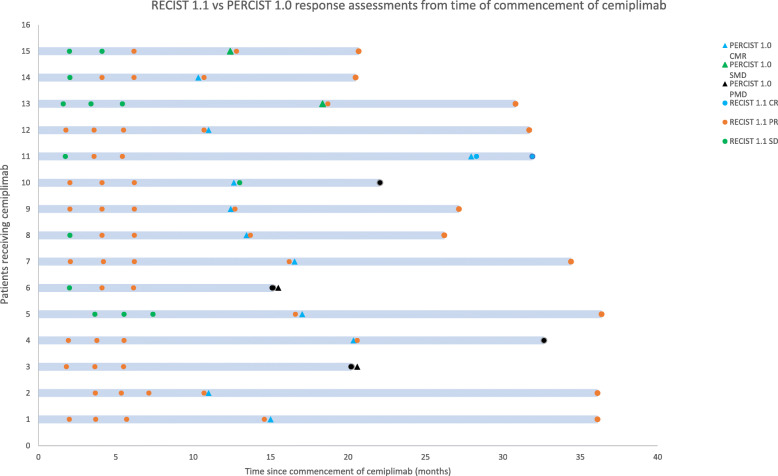
RECIST1.1 and PERCIST1.0 assessments from time of cemiplimab commencement. This figure highlights the relevant response assessments as well as the most recent RECIST1.1 assessment. CMR, complete metabolic response; SMD, stable metabolic disease; PMD, progressive metabolic disease. CR, complete response; PR, partial response. SD, stable disease. PD, progressive disease

Of the 11 patients with CMR, two patients developed disease progression. The first had a PR as per RECIST1.1 and developed small brain metastases that have not required intervention without recurrence in other previous sites 21 months after CMR. The other patient had stable disease as per RECIST1.1 and recurred in regional nodes 9 months post CMR without progression of previous metastases and was referred for surgical resection. Of the two patients with a partial metabolic response, one has an ongoing PR on RECIST1.1 and was thought to also have osteomyelitis, which may have confounded interpretation of the PET imaging. The other patient has ongoing FDG avidity in disease sites at 18 months with ongoing RECIST1.1 PR. Patients who progressed by PERCIST1.0 also progressed by RECIST1.1. Figure 3 highlights the imaging assessments for each patient.

All 15 patients were alive at end of the study follow-up with a median follow-up time of 3.1 years (range 1.74–4.10). The PFS at 3 years was 71.5% [95%CI: 40.4, 88.3].

## Discussion

A pooled analysis after median follow up of 11.1 months of the pivotal phase II cemiplimab study demonstrated a 46% overall response rate (as per RECIST1.1) by independent central review (54% investigator evaluation) [1, 5], with predominantly PRs and an estimated 24 month OS of 73.3% [8]. With an additional year of follow up the CR rates have increased over time from 9 to 16% [6]. We hypothesised that many of these long-term responding patients with measurable disease as per RECIST1.1 criteria did not have active disease, correlating with CMR on FDG-PET/CT. In our cohort, of 11 patients with RECIST1.1 PR, 9 (82%) discordantly had a CMR on FDG-PET/CT in support of this hypothesis.

There are limited data evaluating FDG-PET/CT in CSCC. These consist mostly of case reports and series evaluating its use as a staging tool rather than assessing treatment response [9, 10]. This includes a recent retrospective series in 23 advanced CSCC patients demonstrating high sensitivity in detection of small cutaneous lesions and nodal disease [10] and another retrospective series of 115 patients finding FDG-PET/CT sensitive in detecting recurrent disease leading to a change in management plan for 28% of patients [9]. To the best of our knowledge, our study is the first to review the role of FDG-PET/CT assessment of disease response to immunotherapy in CSCC.

Chemotherapy results in the depopulation of cancer cells in responding patients [11] with the reduction of tumor size and burden reported by RECIST1.1 being the surrogate for clinical benefit and survival [12]. These responses have traditionally been measured by the standardized RECIST1.1 criteria which has been challenged by the use of immunotherapy [11, 12]. Chemotherapy and radiotherapy can cause reduction of measurable disease via direct cytotoxicity. However, with newer therapies including immunotherapy and targeted therapies, a reduction in tumor bulk and size may not necessarily be the best measure of clinical benefit and survival [13]. The immune-related response criteria (irRC), immune-related response evaluation criteria in solid tumors (irRECIST), modified RECIST 1.1 for immunotherapy (iRECIST) and immune-modified RECIST (imRECIST) for CT/MRI have been devised to standardize approaches to tumor assessment in the setting of immunotherapy. However, further prospective data are needed [14–17].

In advanced melanoma, patients achieving a CR as per RECIST1.1 tend to have durable long-term outcomes [4]. However there are a large number of patients with a PR or even SD as per RECIST1.1 that also achieve excellent long term results [18]. This has also been the observation in our cohort of CSCC patients where 67% (9/15) of patients with RECIST1.1 PR had CMR on FDG-PET, all patients were alive at the end of follow up and only two patients experienced progression. Immune-related response criteria may help account for ICI phenomena like pseudoprogression but they fail to identify which patients with a PR or SD may achieve good long-term outcomes [4]. In melanoma patients receiving ICI, the utility of FDG-PET/CT imaging at the 12 month time-point has been prospectively evaluated showing that almost all patients (96%) with a CMR at one year have an ongoing response to therapy thereafter [4]. It has been hypothesised that for those patients with CMR on FDG-PET but with PR or SD as per RECIST1.1, lesions may be representative of scarring rather than active disease [19]. Assessment of cellular metabolism using FDG-PET may be more sensitive for detection of active residual cancer [12, 20]. Limitations of FDG-PET imaging for patients on immunotherapy include potential difficulty in interpretation in the settings of pseudoprogression, immune mediated toxicities or infection. The optimal timing for FDG-PET imaging is also yet to be defined and there are no published data to date on its use in assessing early treatment response in CSCC or guiding early treatment cessation in those achieving a CMR; questions hopefully to be answered by future prospective trials.

## Conclusions

In our cohort of advanced CSCC patients receiving immunotherapy for at least 10 months, the majority of patients with PR as per RECIST1.1 were found to have CMR on FDG-PET/CT. Based in part on these initial observations, prospective serial FDG-PET/CT scans have been incorporated into a subsequent cohort on the NCT02760498 trial, which may help to define the utility of FDG-PET/CT to assess treatment response and prognosis.

## Data Availability

The datasets generated during and/or analysed during the current study are available from the corresponding author on reasonable request.

## Abbreviations

CI: Confidence interval
CSCC: Cutaneous squamous cell carcinoma
CR: Complete response
CMR: Complete metabolic response
CT: Computed tomography
ECOG: Eastern Co-Operative Oncology Group
FDG: Fluorodeoxyglucose
ICI: Immune checkpoint inhibitor
irRC: The immune-related response criteria
irRECIST: Immune-related response evaluation criteria in solid tumors
iRECIST: Modified RECIST 1.1 for immunotherapy
imRECIST: Immune-modified RECIST
MRI: Magnetic resonance imaging
PET: Positron Emission Tomography
PERCIST: PET response criteria in solid tumours
PR: Partial response
PMD: Progressive metabolic disease
RECIST: Response criteria in solid tumours
SD: Stable disease
SMD: Stable metabolic disease
